# Cost‐effectiveness of chemoradiation followed by esophagectomy versus chemoradiation alone in squamous cell carcinoma of the esophagus

**DOI:** 10.1002/cam4.2721

**Published:** 2019-11-20

**Authors:** Jonathan Salcedo, Sze‐chuan Suen, Shelly X. Bian

**Affiliations:** ^1^ Department of Pharmaceutical and Health Economics University of Southern California Los Angeles CA USA; ^2^ Daniel J. Epstein Department of Industrial and Systems Engineering University of Southern California Los Angeles CA USA; ^3^ Leonard D. Schaeffer Center for Health Policy and Economics University of Southern California Los Angeles CA USA; ^4^ Department of Radiation Oncology Keck School of Medicine University of Southern California Los Angeles CA USA

**Keywords:** chemoradiotherapy, cost‐effectiveness analysis, economic evaluation, esophageal squamous cell carcinoma, esophagectomy, Markov process

## Abstract

**Background:**

Standard treatment for locally advanced esophageal cancer usually includes a combination of chemotherapy, radiation, and surgery. In squamous cell carcinoma (SCC), recent studies have indicated that esophagectomy after chemoradiation does not significantly improve survival but may reduce recurrence at the cost of treatment‐related mortality. This study aims to evaluate the cost‐effectiveness of chemoradiation with and without esophagectomy.

**Methods:**

We developed a decision tree and Markov model to compare chemoradiation therapy alone (CRT) versus chemoradiation plus surgery (CRT+S) in a cohort of 57‐year‐old male patients with esophageal SCC, over 25 years. We used information on survival, cancer recurrence, and side effects from a Cochrane meta‐analysis of two randomized trials. Societal utility values and costs of cancer care (2017, USD) were from medical literature. To test robustness, we conducted deterministic (DSA) and probabilistic sensitivity analyses (PSA).

**Results:**

In our base scenario, CRT resulted in less cost for more quality‐adjusted life years (QALYs) compared to CRT+S ($154 082 for 1.32 QALYs/patient versus $165 035 for 1.30 QALYs/patient, respectively). In DSA, changes resulted in scenarios where CRT+S is cost‐effective at thresholds between $100 000‐$150 000/QALY. In PSA, CRT+S was dominant 17.9% and cost‐effective at willingness‐to‐pay of $150 000/QALY 38.9% of the time, and CRT was dominant 30.6% and cost‐effective 61.1% of the time. This indicates that while CRT would be preferred most of the time, variation in parameters may change cost‐effectiveness outcomes.

**Conclusions:**

Our results suggest that more data is needed regarding the clinical benefits of CRT+S for treatment of localized esophageal SCC, although CRT should be cautiously preferred.

## Graphical abstract

We use a Markov model informed by event rates, costs, and utilities from medical literature to show the addition of esophagectomy to definitive chemoradiation is unlikely to be cost‐effective for elderly US males with esophageal squamous cell carcinoma (SCC). However, additional clinical studies on esophagectomy outcomes in SCC are necessary to preclude surgery in all contexts.

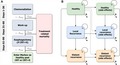

## 1 INTRODUCTION

Esophageal cancer affects approximately 17 000 people and causes over 15 000 deaths annually in the United States. The majority of patients affected are men, and it is the seventh leading cause of cancer death in men in the United States. Due to the lack of serosal layer of the esophagus and no existing screening procedures, cases are often diagnosed at later stages, necessitating multimodality treatment with an average 5‐year overall survival of about 20%.^1^ Squamous cell carcinoma (SCC) is one of the two major histologies of esophageal cancer, making up 90% of cases worldwide and about 35% of cases in the US.

Historically, esophagectomy was the primary treatment for all esophageal cancers. In locally advanced disease (stage II/III), multiple randomized trials in the last 20 years have shown a large survival and local control benefit with the addition of chemoradiation to surgery. This is often given in the neoadjuvant setting prior to esophagectomy, allowing for analysis of pathologic complete response (pCR) rates, which range from 40%‐50% in SCCs. These high pCR rates coupled with high perioperative mortality rates, have led many centers to question the necessity of esophagectomy following chemoradiation.

In the last decade, there have been two major trials evaluating the benefit of the addition of surgery to chemoradiation in esophageal SCC.^2^, ^3^, ^4^ Both trials showed equivalent overall survival with some local control benefit with the addition of surgery. The clinical impact of an endpoint like local control, however, is difficult to interpret in the setting of increased perioperative and possibly long‐term morbidity.

The optimal treatment is therefore controversial and surgery continues to be used in about 30% of locally advanced esophageal SCCs.^5^, ^6^ In the US‐based NCCN guidelines, chemoradiation followed by esophagectomy is still considered standard of care with definitive chemoradiation reserved only for patients who refuse surgery, are medically unfit for resection, or present with cervical esophageal tumors.^7^ However, in Europe, the ESMO guidelines support the routine use of definitive chemoradiation for stage II/III esophageal SCC with close follow‐up.^8^

To date, there have been no cost‐effectiveness analyses specifically looking at the cost or quality of life tradeoffs associated with the addition of esophagectomy to chemoradiation. We use data from the two main randomized trials as well as a Cochrane review to perform a cost‐effectiveness analysis from the US healthcare sector perspective. We also aim to use our data to identify situations in which one treatment paradigm would be preferred over the other.

## 2 METHODS

### 2.1 Model overview

We developed a decision tree and Markov transition state model in Excel (Microsoft Corporation) to compare treatment with chemoradiation alone (CRT) versus chemoradiation plus surgery (CRT+S) in a cohort of 57‐year‐old male patients with squamous cancer of the esophagus. A Markov model is a commonly used tool to simulate the movement of patients between health states over time, allowing us to calculate the total discounted lifetime costs and health outcomes for each simulated patient. The probability of transition between health states in the model reflect empirically observed rates of transition.

We selected treatment arms to reflect common treatment strategies in male patients. We chose 57‐year‐old males to reflect the most typical patient observed in two randomized controlled trials (RCTs).^3^, ^4^ Information on survival, cancer recurrence, and side effects were taken from a Cochrane meta‐analysis of two randomized trials examining chemoradiation with and without esophagectomy.^2^, ^3^, ^4^ Utility values by health state and costs of cancer care were extracted from the medical literature. The model horizon was 25 years with monthly cycles, including a 3‐month treatment phase beginning at diagnosis and ending at treatment completion, for both arms. All costs are reported in 2017 USD and outcomes were evaluated from the United States healthcare sector perspective. Both costs and quality‐adjusted life years (QALYs) were discounted at the same rate of 3% per year.^9^ The primary model outcome of interest was the incremental cost‐effectiveness ratio (ICER) between CRT and CRT+S. An ICER indicates the additional cost incurred to obtain one additional QALY.

### 2.2 Health states

**Figure 1:**
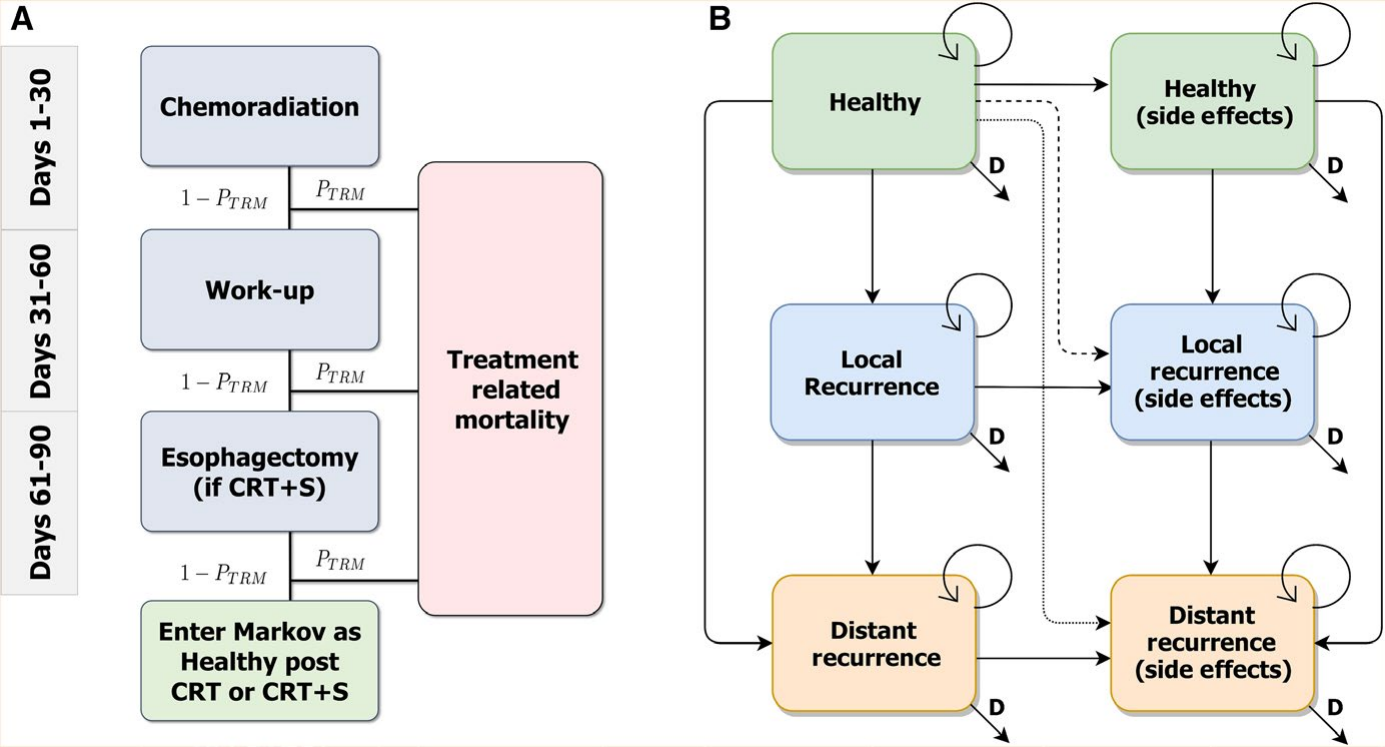
Health states and transitions. A, Decision tree of treatment phase (initial 90 days, both arms); B, Markov model post‐CA or post‐CRT+S. CRT, chemoradiation‐alone; CRT+S, chemoradiation plus surgery; TRM, treatment‐related mortality; D, death

The model follows individuals over time as they move between health states. Possible health states in the model are generated from information available in RCTs for squamous cancer of the esophagus.^2^, ^3^, ^4^ In the first month after diagnosis, we assume the patient will receive standard chemotherapy and radiation. In the 30 days after chemoradiation, patients in both arms receive work‐up and the CRT arm receive additional chemoradiation. In the final 30 days of the treatment phase, the CRT arm receive final chemotherapy treatment and the CRT+S arm undergoes esophagectomy. At the end of the 90 days, surviving patients continue in the model as healthy, post‐CRT or post‐CRT+S treatment. We intend for our efficacy estimates to reflect modern treatment procedures; however, we are limited to the most recent RCT evidence available (the treatment regimens described in Stahl et al and Bedenne et al). See Figure 1A for the decision tree.

After the initial treatment phase, patients from either treatment arm may transition between health states (see Figure 1B). The health states include healthy, local recurrence, and distant recurrence – each with and without side effects. Transition probabilities, including death rates during the first four years, are dependent on treatment arm. Death rates after four years are from US life tables.^10^ See Table 1 for a summary of transition probabilities and Data S1 for additional details.

**Table 1:** Baseline model parameters

Description	Years	CRT	CRT+S	Source
Baseline probabilities				
Treatment‐related mortality	3‐month	0.0052	0.0926	[4]
Local recurrence, monthly	1 and 2	0.0237	0.0208	Calculated, [3]
	3 and 4	0.0237	0.0000	Calculated, [3]
	5 onward	0.0000	0.0000	Assumption, [3]
Distant recurrence, monthly	All years	0.0142	0.0205	Calculated, [4]
Side effects, monthly	All years	0.0081	0.0012	Calculated, [4]
Death, monthly	1 to 4	Varies	Varies	[4]
	5 onward	Life table	Life table	[10]
Utilities (QALY weights)				
Treatment	3 months	0.770	0.300	[16]‐[18]
Healthy	All years^a^	0.770	0.727	[16, 17, 19, 20]
Local recurrence	All years^a^	0.460	0.417	[16, 17, 19, 20]
Distant recurrence	All years^a^	0.150	0.107	[16, 17, 19, 20]
Side effects (decrement)	All years^a^	‐0.350	‐0.350	[16]
Death	All years^a^	0.000	0.000	—
Total costs				
Initial phase	First year	$94 128	$133 290	Calculated, [11, 13, 14]
Continuing phase	Middle years	$7893	$7893	[11]
End‐of‐life phase	Last year	$126 959	$126 959	[11]

Abbreviations: CRT, chemoradiation‐alone; CRT+S, chemoradiation plus surgery; QALY, quality‐adjusted life year.

a Utilities for both arms were assumed to be equal after 12 months, ie utility in health state *i* equals *max (util_i,CRT_,util_i,CRT+S_)*.

### 2.3 Costs

We estimated costs for patients in each health state from a published study of the Surveillance, Epidemiology, and End Results (SEER) Medicare‐linked claims through 2006.^11^ This study provided estimated costs of treatment for esophageal cancer in the initial (first twelve months), final (last twelve months), and continuing (any remaining months) phases of life. If patients die within twelve months of diagnosis, months are counted as final rather than initial, as is done in prior studies of cancer costs.^11^, ^12^

No empirical studies provided separate cost estimates of esophagectomy, chemoradiation, and esophagectomy plus chemoradiation. We therefore calculated differences in costs during the initial treatment phases by treatment arm by decomposing average total cost provided in the literature.^11^, ^13^, ^14^ We adjusted all costs to 2017 US dollars using the medical care services component of the consumer price index (CPI).^15^ A summary of final costs by treatment arm and phase of life is provided in Table 1. See Data S1 for additional costing details.

### 2.4 Utilities

We evaluate all health benefits in QALYs. Values for being healthy, in local recurrence, or in distant recurrence, post‐CRT are sourced from the literature. We assumed side effects resulted in a QALY loss of 0.350 from the underlying health state.^16^ We extracted the short‐term utility (0.300) and long‐term decrement of esophagectomy treatment (−0.043) from previous cost‐effectiveness analyses.^17^, ^18^, ^19^, ^20^ Consistent with health‐related quality of life (HR‐QoL) findings in Bonnetain et al, we assumed identical utility values for both arms after one year (duration varied in sensitivity analysis).^21^ See Table 1 for a summary of utility values by treatment arm and Data S1 for details.

### 2.5 Sensivity analyses

To check for robustness of model results, we conducted deterministic (DSA) and probabilistic sensitivity analyses (PSA). In addition to reporting ICERs, we find CRT incremental net monetary benefits (INMB) with willingness to pay (WTP) of $150 000 per QALY.^22^ For one‐way and two‐way DSA, we varied model inputs within their 95% confidence intervals (CI), or ±50% of the base case when the 95% CI was not available. In two‐way sensitivity analyses, we varied pairs of parameters found to affect model results in one‐way DSA. In PSA, we fit distributions to input parameters and simulated outcomes in 10 000 Monte Carlo repetitions.^23^ We used typical distributions for each parameter type (see Data S1 for details).

## 3 RESULTS

In the base case analysis, chemoradiation alone resulted in less cost for more QALYs compared to chemoradiation plus surgery ($154 082 for 1.32 QALYs/patient versus $165 035 for 1.30 QALYs/patient, respectively). This results in CRT being the dominant treatment in the base case. Due to a dominant base case result we instead report INMB, as a negative ICER is not meaningful. At a WTP threshold of $150 000 per QALY, this results in an INMB of $13 862 for CRT over CRT+S.

### 3.1 Deterministic sensitivity analyses

**Figure 2:**
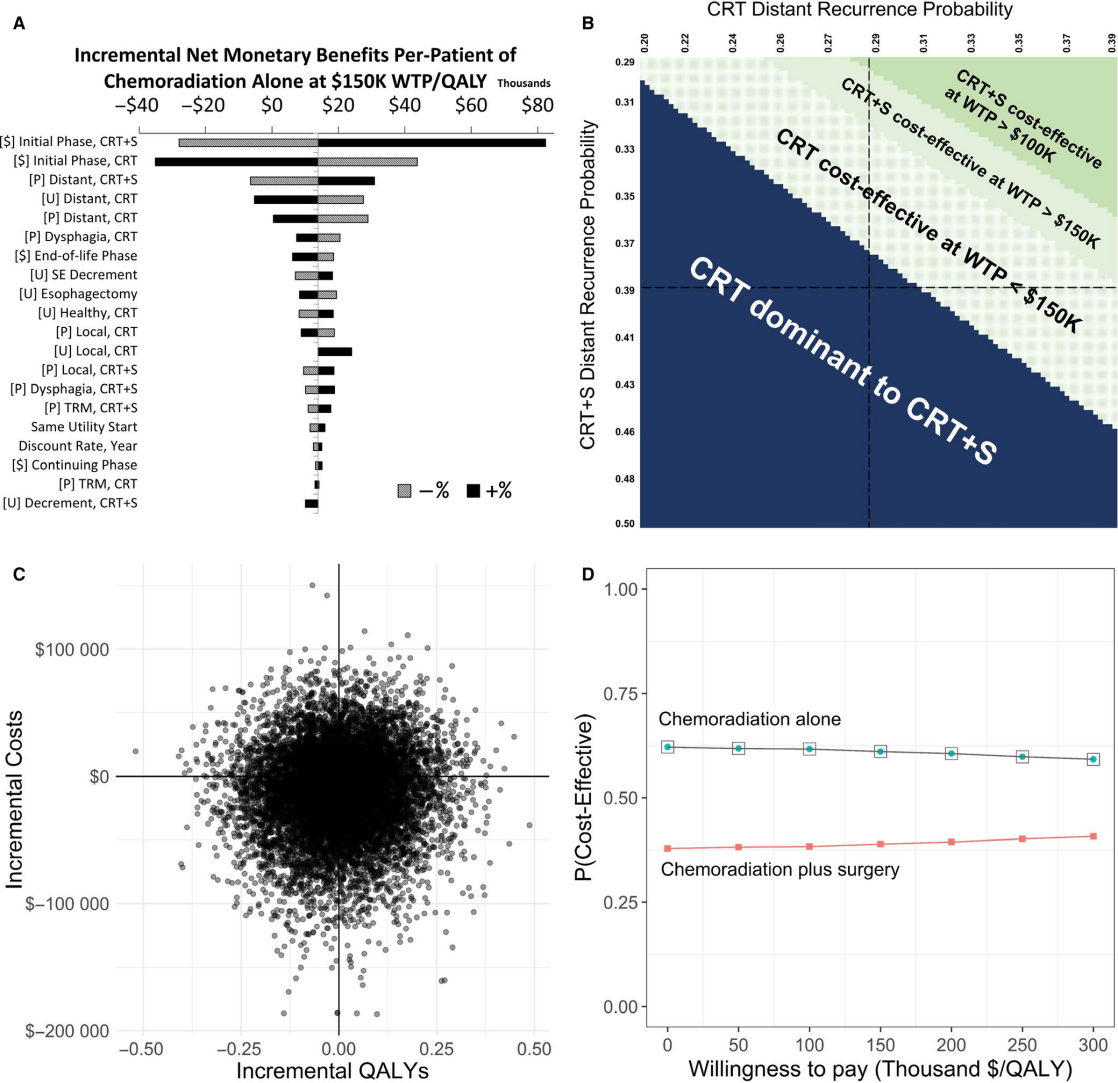
Deterministic and probabilistic sensitivity analyses. A, Tornado diagram for INMB of CRT relative to CRT+S; B, Two‐way DSA of distant recurrence probabilities; C, Incremental costs/QALYs of CRT relative to CRT+S from PSA (10 000 iterations); D, Cost‐effectiveness acceptability curve (CEAC) and frontier (CEAF). CRT, chemoradiation alone; CRT+S, chemoradiation plus surgery; WTP, willingness to pay; QALY, quality‐adjusted life year; SE, side effects; TRM, treatment‐related mortality; P, probability; U; utility

The input parameters used in our base case model may vary across hospitals or regions, and we explored model results if single input values were varied between its 95% confidence intervals reported in the literature while holding all other values constant. We found that CRT+S would become cost‐effective with a WTP of $150K if CRT+S initial phase costs were $101 129 (at most $7001 more than the CRT initial phase cost), the monthly probability of annual recurrence in CRT+S was as low as 0.016, or in CRT as high as 0.020. Increasing the utility of the distant recurrence state to 0.286 resulted in an ICER for CRT+S of $101 734. These analyses suggest that univariate changes to the model may result in scenarios where CRT+S is cost‐effective at thresholds between $100 000 and three times the USA GDP per capita. See Figure 2A for a tornado diagram of all parameters.

While the base case analysis uses the mean reported two‐year probabilities of distant recurrence (0.391 for CRT+S and 0.291 for CRT), if the two values were identical, CRT+S would be cost‐effective for WTP thresholds of $100k or $150k (see Figure 2B), indicating that uncertainty in the reported literature can substantially change our base case results.

### 3.2 Probabilistic sensitivity analyses

To explore how our results change when we vary many inputs at once, we perform PSA (see Figure 2C). We find that CRT+S was dominant 17.9% of the time and cost‐effective at WTP of $150K/QALY compared to CRT 38.9% of the time, indicating that uncertainty in the input parameters may lead to CRT+S being preferred over CRT in some cases. However, despite this uncertainty, CRT will be the recommended strategy the majority of time, as it was dominant 30.6% of the time and cost‐effective at WTP of $150K/QALY 61.1% of the time. This does not vary much by WTP; as we increased WTP from $50K to $300K/QALY, CRT+S was only 2.6 percentage points more likely to be cost‐effective (see Figure 2D).

These sensitivity analysis results indicate that while chemoradiation alone would be preferred most of the time, variation in inputs may change cost‐effectiveness outcomes.

## 4 DISCUSSION

We find that chemoradiation alone dominates chemoradiation with surgery for 57‐year‐old males with SCC of the esophagus. Prior literature has found esophagectomy statistically significantly decreases locoregional progression and palliative interventions for dysphagia.^4^ However, we find these benefits unlikely to improve health‐related quality of life enough to outweigh drawbacks of the procedure (costs and TRM). Paired with the additional financial expense incurred by the patient and healthcare system, our model suggests the addition of esophagectomy is unlikely to be cost‐effective. In DSA, monetary benefits of CRT over CRT+S were sensitive to the probability of dysphagia for CRT patients, but not enough to change our base case outcome.

Factors that could unilaterally change the base result of our model included initial year costs as well as probabilities and utilities of distant (metastatic) recurrence. While changes in costs could result in CRT being cost‐prohibitive, it is likely that CRT costs are less than CRT+S costs for most healthcare providers in the US. Our model is sensitive to changes in distant recurrence and may result in an ICER of <$150 000 for CRT+S. Given differences in distant recurrence probability were not statistically significant in RCT evidence, it is possible that CRT+S treatment results in slightly more QALYs than CRT and is cost‐effective at WTP of three times USA GDP per capita.^4^

Current evidence shows that there are clinical trade‐offs for esophagectomy in patients with SCC treated with chemoradiation. Patients who survive the procedure may benefit from decreased risks of all‐cause mortality, locoregional progression, and high‐grade dysphagia. However, treatment‐related mortality can be substantial and vary depending on treatment setting and patient population.^24^, ^25^ Post‐operative mortality may vary and be particularly low in high‐volume centers. Patients treated in high‐volume hospitals with low treatment‐related mortality may find the treatment benefits outweigh the risk of death. We therefore include post‐operative mortality in our sensitivity analysis. Variation to the documented 95% confidence intervals on this value does not change our finding on the cost‐effectiveness of CRT.^2^, ^3^, ^4^ Esophagectomy may also be attractive to patients who are younger with fewer comorbidities and hence more likely to survive the procedure. These factors are likely to influence cost‐effectiveness results and support the need for clinicians to practice patient‐centered care in treating esophageal cancer.

We acknowledge several limitations of this study. Distant recurrence rates were taken from Bedenne et al and, although numerically different, were not statistically different in the two arms.^4^ We find model results to be sensitive to these inputs, suggesting cost‐effectiveness outcomes may change based on uncertainty in values from the literature. As Bedenne et al found statistically significant differences in palliative interventions for dysphagia, our model uses stent interventions for dysphagia as a proxy for all chronic side effects. Other side effects of chemoradiation can include strictures, fistula, and lung toxicity; however, rates of these complications may be similar across treatment arms. Our study cohort is males of mean age 57, which limits generalizability of our base case results to different age groups or genders. However, in the United States the male to female ratio of the disease approaches 4:1, and incidence peaks in the sixth decade of life.^26^

Lastly, we are limited to the treatment regimens described in Stahl et al and Bedenne et al for efficacy estimates, since they are the most recent RCT evidence available for our comparison in esophageal SCC.^2^, ^3^, ^4^ Other data suggest radiation dose escalation may increase resource utilization in the CRT arm without improving survival or local/regional control.^27^ Hence, our base case may slightly underestimate the cost‐effectiveness of CRT relative to CRT+S in modern practice where dose escalation is uncommon. We intend for the model to reflect generally accepted treatment procedures but are limited by the latest information available in the literature.

Our results indicate that more data is needed on clinical benefits of CRT+S for treatment of localized esophageal carcinoma, although CRT should be cautiously preferred. While a recent review has shed light on existing RCT evidence for trimodal therapy (CRT+S) versus bimodal therapy (CRT), no randomized studies have been conducted within the past ten years. Advances in health technology in recent years have likely resulted in safer treatment conditions and improved surgical methods. Evidence‐based practice in cancer treatment is heavily reliant on new research to determine best course of treatment. We hope additional studies on the efficacy, safety, and costs of chemoradiation plus surgery are forthcoming.

## CONFLICT OF INTEREST

The authors have declared no conflicts of interest.

## AUTHOR CONTRIBUTIONS

Jonathan Salcedo: Methodology, software, formal analysis, investigation, data curation, visualization, writing – original draft preparation, and writing—review and editing. Sze‐chuan Suen: Conceptualization, methodology, supervision, project administration, formal analysis, writing—original draft preparation, and writing—review and editing. Shelly Xiaolu Bian: Conceptualization, methodology, supervision, project administration, resources, writing—original draft preparation, and writing—review and editing.

## Data Availability

The data that support the findings of this study are available from the corresponding author upon reasonable request.
