# Supplementary material for: Cost‐effectiveness of chemoradiation followed by esophagectomy versus chemoradiation alone in squamous cell carcinoma of the esophagus
Source: Cancer Med. 2019 Nov 20;9(2):440–6. doi: 10.1002/cam4.2721 (PMC6970052; doi:10.1002/cam4.2721)
Supplement: Supplementary file 1 [file CAM4-9-440-s001.docx]

**Supporting Information: Cost-Effectiveness of Chemoradiation followed by Esophagectomy Versus Chemoradiation Alone in Squamous Cell Carcinoma of the Esophagus.**

**Health states**

In the first month after diagnosis, we assume the patient will receive standard chemotherapy and radiation. Radiation is assumed to be either split-course or conventional. In the thirty days after chemoradiation, patients in both arms receive work-up. From the RCT Bedenne et al., work-up involves “clinical examination, gastroscopy with biopsies, esophagogram, bronchoscopy, supraclavicular ultrasonography, thoracoabdominal CT scan, and endoscopic ultrasonography when available.”^1^ In the final thirty days of the treatment phase the CRT+S arm undergoes esophagectomy. We chose cycles of thirty days and an estimated total treatment duration of ninety days to mirror the treatment patterns observed in Bedenne et al.^1^ At the end of the ninety days, surviving patients continue in the model as healthy, post-CRT or post-CRT+S treatment. Table 1 in the main text provides these transitions.

After the initial treatment phase, patients from either treatment arm may transition between health states (see main text Figure 1B). The health states include healthy, local recurrence, and distant recurrence – each with and without side effects – to capture the likelihood of cancer recurrence across their remaining lifetimes. Probabilities of developing recurrence or chronic side effects are dependent on treatment arm. Rates of death specific to treatment arm for the first four years post-diagnosis are from digitized RCT data (see Figure S1).^1,2^

**Figure S1. Digitized mortality curves from Bedenne et al. (2007).**



CRT, chemoradiation alone; CRT+S, chemoradiation plus surgery.

Following evidence from the RCT documented in Stahl et al., we assumed no new cases of local recurrence after two years for the CRT+S arm and after four years for the CRT arm.^3^ Based on Stahl et al. and expert clinical opinion, we assumed probability of death to be equivalent to US male baseline mortality after four years in both arms.^3,4^ See Table 1 in the main text for a summary of transition probabilities including changes over time.

**Transition probability calculations**

We calculated transition probabilities in the model using event rates in two published randomized controlled trials of esophagectomy plus chemoradiation versus chemoradiation alone for the treatment of esophageal cancer.^1,3,5^ We digitized survival data for each arm from the more recent RCT (Bedenne et al.) to provide empirical death rates for the first four years.^1,2^ Based on the other RCT (Stahl et al.) and expert clinical opinion, we assumed patients surviving past four years had mortality rates similar to the general population.^3,4^ See Figure S2 for the Markov trace by treatment arm.

**Figure S2. Markov trace by treatment arm.**





Deterministic progression of 1,000 theoretical patients over model horizon of 25 years, monthly cycles. CRT, chemoradiation alone; CRT+S, chemoradiation plus surgery.

For other events in the model, we took point estimates from the clinical trials. We extracted two-year probability of locoregional progression for surgery patients (0.396) and four-year probability of locoregional progression for non-surgery patients (0.683) from Stahl et al.^3^ We extracted two-year probability of distant recurrence for surgery (0.391) and non-surgery patients (0.290) from Bedenne et al.^1^ We approximated likelihood of chronic side-effects using four-year probabilities of stent intervention for dysphagia from Bedenne et al. which were 0.054 and 0.323 for surgery and non-surgery arms, respectively.^1^ We assumed these probabilities were generated from cumulative exponential distributions which allowed us to generate transition probabilities for monthly cycles using the following relationship.^6,7^

r = -ln(1-p)/t

p = 1-exp(-rt)

**Estimation of costs and utilities**

We estimated total monthly costs of cancer based on the patient length of survival and initial treatment received. Since no empirical source in the medical literature provided total healthcare cost burden of esophageal cancer patient by treatment, we calculated model costs using multiple published studies.

Mariotto et al. provided estimated costs for elderly esophageal cancer patients based on phase of care: initial, continuing, or end-of-life from an analysis of SEER-Medicare data.^8^ They defined the initial, end-of-life, and continuing phases as: the first 12 months following diagnosis, the last 12 months of life, and any months in between, respectively. For patients surviving fewer than 24 months, they allocated final months to end-of-life with any remaining months allocated to the initial phase. For example, a patient surviving 17 months following diagnosis would be counted as having 5 months of initial and 12 months of end-of-life care. This definition has been used previously in the literature.^9,10^

We took costs for the continuing and end-of-life phases in the model directly from Mariotto et al. and adjusted to 2017 figures using the medical component of the US consumer price index (CPI).^8,11^ Estimating costs for the initial treatment phase involved decomposing the value provided in Mariotto et al. to account for heterogeneity based on treatment received. One study, Worni et al., studied treatment patterns for esophageal cancer patients in the US across 1998 to 2008 using SEER data. Among 3,295 treated patients, they observed bi-modal treatment (radiation and surgery) in 38.7%, surgery-only treatment in 21.1%, and radiation-only treatment in 40.2%.^12^ We used these treatment distributions in addition to an estimated cost of esophagectomy provided by Pohl et al. ($39,162 when adjusted to 2017 USD) to calculate the initial phase costs in our model: $133,290 for chemoradiation plus surgery and $94,128 for chemoradiation-alone in the first year following diagnosis.^13^ We adjusted all costs to 2017 US dollars using the medical care services component of the consumer price index (CPI).^11^

We evaluate all health benefits in quality adjusted life years (QALYs), which are calculated by multiplying each state’s utility value by the number of years spent in the state. Each state’s utility value is based on the expected quality of life while in that state. We sourced baseline utilities from Wildi et al. which provides societal time trade-off (TTO) utilities for patients with esophageal cancer and varying levels of dysphagia.^14^

**Sensitivity analysis**

In probabilistic sensitivity analysis, we used typical distributions for various parameter types to characterize uncertainty. We fit costs to gamma distributions to model positive skewness commonly observed in empirical healthcare cost data.^15^ We conservatively assumed unknown standard errors were 50% of their mean cost. We modeled event probabilities with beta distributions and fit using the method of moments or effective sample sizes when standard errors were unavailable.^16,17^ For utilities, or QALY weights, we fit these to beta distributions when standard errors or statistical parameters were available in the medical literature. All remaining parameters were fit to uniform distributions to reflect uninformative prior distributions. Figure S3 shows each varied parameter and the respective histogram of PSA draws. Table S1 details input parameters’ distributions and respective parameters.

**Figure S3. Histograms of Monte Carlo PSA draws by parameter.**





Results of 10,000 iterations, 100 bins. CRT, chemoradiation alone; CRT+S, chemoradiation plus surgery; $, 2017 USD (thousands); P, probability; U; utility.

**Table S1. Distributions and parameters used in probabilistic sensitivity analysis**

| **Input** | **Arm** | **Distribution** | **Parameter 1** | **Parameter 2** |
| --- | --- | --- | --- | --- |
| **General Parameters** |  |  |  |  |
| Discount rate, yearly | Both | Uniform(a,b) | 0.02 | 0.05 |
| **Source Probabilities** |  |  |  |  |
| Treatment related mortality, three-month | CRT+S | Beta(α,β) | 10.28 | 100.72 |
|  | CRT | Beta(α,β) | 0.73 | 141.27 |
| Locoregional progression, two-year | CRT+S | Beta(α,β) | 29.68 | 45.32 |
| Locoregional progression, four-year | CRT | Beta(α,β) | 55.34 | 25.66 |
| Distant progression, two-year | CRT+S | Beta(α,β) | 32.75 | 51.02 |
|  | CRT | Beta(α,β) | 26.74 | 65.47 |
| Side-effects, four-year | CRT+S | Beta(α,β) | 7.00 | 122.00 |
|  | CRT | Beta(α,β) | 42.00 | 88.00 |
| **Source Utilities** |  |  |  |  |
| Treatment | CRT+S | Uniform(a,b) | 0.15 | 0.45 |
|  | CRT | Beta(α,β) | 25.27 | 7.55 |
| Healthy | CRT | Beta(α,β) | 25.27 | 7.55 |
| Locoregional progression | CRT | Beta(α,β) | 21.93 | 25.74 |
| Distant progression | CRT | Beta(α,β) | 5.05 | 28.64 |
| Esophagectomy, decrement | CRT+S | Beta(α,β) | 4.00 | 88.30 |
| Side-effects, decrement | Both | Uniform(a,b) | -0.61 | -0.24 |
| **Total Costs** |  |  |  |  |
| Initial phase | CRT+S | Gamma(α,θ) | 4.00 | 33322.46 |
|  | CRT | Gamma(α,θ) | 4.00 | 23531.90 |
| Continuing phase | Both | Gamma(α,θ) | 4.00 | 1973.37 |
| End-of-life phase | Both | Gamma(α,θ) | 4.00 | 31739.71 |

CRT, chemoradiation alone; CRT+S, chemoradiation plus surgery.

**References**

1. Bedenne L, Michel P, Bouche O, et al. Chemoradiation followed by surgery compared with chemoradiation alone in squamous cancer of the esophagus: FFCD 9102. *J Clin Oncol.* 2007;25(10):1160-1168.

2. Rohatgi A. WebPlotDigitizer 4.0. 2017; https://automeris.io/WebPlotDigitizer. Accessed November 2, 2017.

3. Stahl M, Stuschke M, Lehmann N, et al. Chemoradiation with and without surgery in patients with locally advanced squamous cell carcinoma of the esophagus. *J Clin Oncol.* 2005;23(10):2310-2317.

4. Arias E, Heron M, Xu JQ. United States Life Tables, 2012. *Natl Vital Stat Rep.* 2016;65(8):1-64.

5. Vellayappan BA, Soon YY, Ku GY, Leong CN, Lu JJ, Tey JC. Chemoradiotherapy versus chemoradiotherapy plus surgery for esophageal cancer. *Cochrane Database Syst Rev.* 2017;8:Cd010511.

6. Beck JR, Pauker SG, Gottlieb JE, Klein K, Kassirer JP. A convenient approximation of life expectancy (the "DEALE"). II. Use in medical decision-making. *Am J Med.* 1982;73(6):889-897.

7. Beck JR, Kassirer JP, Pauker SG. A convenient approximation of life expectancy (the "DEALE"). I. Validation of the method. *Am J Med.* 1982;73(6):883-888.

8. Mariotto AB, Yabroff KR, Shao Y, Feuer EJ, Brown ML. Projections of the cost of cancer care in the United States: 2010-2020. *J Natl Cancer Inst.* 2011;103(2):117-128.

9. Mariotto AB, Yabroff KR, Feuer EJ, De Angelis R, Brown M. Projecting the number of patients with colorectal carcinoma by phases of care in the US: 2000-2020. *Cancer Causes Control.* 2006;17(10):1215-1226.

10. Yabroff KR, Lamont EB, Mariotto A, et al. Cost of care for elderly cancer patients in the United States. *J Natl Cancer Inst.* 2008;100(9):630-641.

11. U.S. Bureau of Labor Statistics. Medical care in U.S. city average. *CPI-All Urban Consumers (Current Series)* 2017; https://data.bls.gov/timeseries/CUUR0000SAM?output_view=pct_12mths. Accessed August 1, 2017.

12. Worni M, Castleberry AW, Gloor B, et al. Trends and outcomes in the use of surgery and radiation for the treatment of locally advanced esophageal cancer: a propensity score adjusted analysis of the surveillance, epidemiology, and end results registry from 1998 to 2008. *Dis Esophagus.* 2014;27(7):662-669.

13. Pohl HS, A.; Strobel, S.; Eckardt, A.; Rosch, T. Endoscopic versus surgical therapy for early cancer in Barrett's esophagus: a decision analysis. *Gastrointest Endosc.* 2009;70(4):623-631.

14. Wildi SM, Cox MH, Clark LL, et al. Assessment of health state utilities and quality of life in patients with malignant esophageal Dysphagia. *Am J Gastroenterol.* 2004;99(6):1044-1049.

15. Mihaylova B, Briggs A, O'Hagan A, Thompson SG. Review of statistical methods for analysing healthcare resources and costs. *Health Econ.* 2011;20(8):897-916.

16. Briggs AH, Claxton K, Sculpher MJ. *Decision modelling for health economic evaluation.* Oxford: Oxford University Press; 2006.

17. Gelman A. *Bayesian data analysis.* Third edition. ed. Boca Raton: CRC Press; 2014.
